# Targeted Neonatal Echocardiography in Neonatal Patent Ductus Arteriosus Management: A Systematic Review of Clinical Utility and Outcomes

**DOI:** 10.3390/medicina61081442

**Published:** 2025-08-11

**Authors:** Hassan Al-shehri

**Affiliations:** Department of Pediatrics, College of Medicine, Imam Mohammad Ibn Saud Islamic University (IMSIU), Riyadh 13317, Saudi Arabia; haalshehri@imamu.edu.sa

**Keywords:** patent ductus arteriosus, targeted neonatal echocardiography, neonates, efficacy

## Abstract

*Background and Objectives*: Patent ductus arteriosus (PDA) is one of the most common cardiovascular conditions affecting preterm infants, with incidence rates reaching 60% in neonates born before 28 weeks gestation. Traditional clinical assessment alone often proves inadequate for accurate diagnosis, potentially leading to both overtreatment and undertreatment. Targeted neonatal echocardiography (TnECHO) has emerged as a powerful bedside tool that enables neonatologists to perform focused cardiac evaluations, providing real-time assessment of ductal significance and systemic hemodynamics. This systematic review aimed to comprehensively evaluate the clinical utility of TnECHO in the management of PDA in preterm infants, with specific focus on its diagnostic accuracy, impact on treatment decisions, and influence on clinical outcomes. *Materials and Methods*: Following PRISMA guidelines, we conducted a systematic search of PubMed, Web of Science, and Scopus from inception (earliest available date of each database) through February 2025. The search strategy combined terms for “Targeted Neonatal Echocardiography” and “Patent Ductus Arteriosus.” We included observational studies and randomized controlled trials (RCTs) evaluating TnECHO in PDA management, while excluding reviews and case reports. Data extraction focused on study design, population characteristics, TnECHO protocols, and clinical outcomes. *Results*: From 173 initial records, 11 studies met inclusion criteria. Eight studies were rated as high-quality (NOS score ≥ 7). TnECHO implementation was associated with a 49% reduction in PDA ligation rates and decreased need for multiple treatment courses. Studies demonstrated improved diagnostic precision in assessing shunt significance and myocardial function, leading to more tailored therapeutic approaches. The establishment of dedicated TnECHO services enhanced interdisciplinary collaboration between neonatologists and cardiologists. However, limitations included operator dependence, variable institutional protocols, and occasional missed minor cardiac anomalies. *Conclusions*: TnECHO represents a transformative approach to PDA management in preterm infants, enabling physiology-guided decision-making that reduces unnecessary interventions while maintaining patient safety. Current evidence supports its role in improving diagnostic accuracy and optimizing treatment timing. Future research should prioritize multicenter RCTs to establish standardized protocols and evaluate long-term neurodevelopmental outcomes. The integration of TnECHO into routine neonatal practice requires investment in training programs and quality assurance measures to maximize its clinical potential.

## 1. Introduction

Patent ductus arteriosus (PDA) is a common problem in premature infants within the neonatal critical care unit. The prevalence of PDA is inversely correlated with gestational age, ranging from 20% in newborns born at 32 weeks to nearly 60% in those born at less than 28 weeks of gestation [1]. While pharmacological intervention with non-steroidal anti-inflammatory drugs (NSAIDs) or acetaminophen is presently regarded as the primary treatment for PDA, there exists a lack of consensus on the definition of hemodynamically significant PDA and a standardized therapeutic protocol [2,3]. The discourse regarding the association between PDA and several infant morbidities has resulted in a more conservative strategy for PDA management [4,5,6].

Targeted neonatal echocardiography (TnECHO) denotes a bedside, purpose-driven, restricted heart evaluation conducted by a neonatologist to address a particular clinical inquiry [7,8,9]. Clinical disorders in which TnECHO has shown beneficial in directing clinical care encompass PDA, cardiovascular instability, chronic pulmonary hypertension, and congenital diaphragmatic hernia. The most recent 2024 guidelines from the American Society of Echocardiography provide comprehensive updates on the clinical indications, implementation strategies, and training recommendations for TnECHO in neonatal care [10]. These guidelines serve as a critical benchmark for integrating TnECHO into clinical practice. The evaluation of the PDA is the predominant reason for soliciting TnECHO [11,12,13]. The incidence of PDA in preterm neonates is from 15 to 40%, but it ranges from 50 to 65% in very low birth weight neonates (weighing less than 1000 g).

Relying solely on clinical assessment to ascertain the clinical relevance of a PDA may result in erroneous conclusions and unwarranted treatment measures. Echocardiography is the preferred diagnostic method for evaluating the presence or absence of PDA [14,15]. In recent decades, a body of research has developed that presents specific echocardiographic characteristics indicating the hemodynamic relevance of PDA [16,17]. The integration of clinical observations with echocardiographic measures offers a logical approach to assessing the necessity for treatment [18,19]. In the absence of a TnECHO program, choices about PDA treatment are either clinically driven or reliant on echocardiographic data from pediatric cardiologists. With the advancement of TnECHO, trained neonatologists can employ TnECHO to make decisions regarding PDA treatment. A TnECHO-based hemodynamic consultation service was established in our center in 2018. Since its inception, PDA treatment is predicated on the suggestions of the TnECHO team, which integrates clinical results with echocardiographic information. Previously, the management of PDA relied solely on clinical judgment or was informed by consultations with pediatric cardiologists, who evaluated and interpreted conventional pediatric echocardiograms performed by certified sonographers [20,21,22,23]. In the current systematic review, we aimed to investigate the different uses of TnECHO in neonates with PDA and to investigate the effects of its use on the outcomes of PDA management.

## 2. Methods

At every stage, following the Cochrane Handbook of Systematic Reviews of Interventions [24], we performed this systematic review in accordance with the Preferred Reporting Items for Systematic Reviews and Meta-Analyses (PRISMA) statement’s recommendations [25].

### 2.1. Database Searching

PubMed, Web of Science, and Scopus were searched from inception (earliest available date of each database) to February 2025 utilizing the following search strategy: “Targeted Neonatal Echocardiography” and “Patent ductus Arteriosus”. Relevant publications were searched to go through screening in order to be considered for inclusion in our study. Title and abstract filters were used in PubMed, abstract in Web of Science and title, abstract, and keywords in Scopus.

### 2.2. Screening

Following database searching, EndNote version 7 software was used to eliminate duplicates from the generated articles [26]. Then, the rest of the content was transferred to Rayyan software to carry out the screening procedure [27]. Rayyan was used to facilitate blinded screening of abstracts and full-text articles. Although Rayyan includes machine learning features, all inclusion and exclusion decisions were made manually. The included papers were first screened by title and abstract to determine their eligibility for inclusion, and then they were screened using the full text of the included articles from the first phase.

### 2.3. Inclusion and Exclusion Criteria

Any observational or randomized controlled trials (RCTs) investigating the use of TnECHO in neonates with PDA. Reviews and case reports were not included. Studies that investigated non-neonatal populations, non-cardiac indications, or interventions unrelated to TnECHO (e.g., pharmacologic treatments without echocardiographic guidance, or imaging modalities other than echocardiography) were excluded.

### 2.4. Quality Assessment

The quality of the included observational cohort studies was assessed utilizing the Cochrane New Castle Ottawa Scale (NOS) tool. Except for the comparison question, which may earn two stars, each of the eight questions has a maximum score of one star. Consequently, nine represents the maximum attainable score, while zero denotes the minimum. Studies scoring 0–3 were classified as low quality, those scoring 4–6 as fairly excellent, and those scoring 7–9 as highly good [28].

### 2.5. Data Extraction

The baseline data were extracted including study design, population sample size, age, and gender in addition to the aim of each study and the summary of findings provided by each of them. This was performed using Microsoft Excel sheets.

### 2.6. Qualitative Synthesis

A review of the literature was performed to encapsulate conclusions from the research that was incorporated, concentrating on the effectiveness of use of TnECHO in patients with PDA. Data in the included studies were analyzed based on different outcomes related to the use of TnECHO and providing further insight into the benefits of use.

## 3. Results

### 3.1. Database Searching and Screening

**Figure 1:**
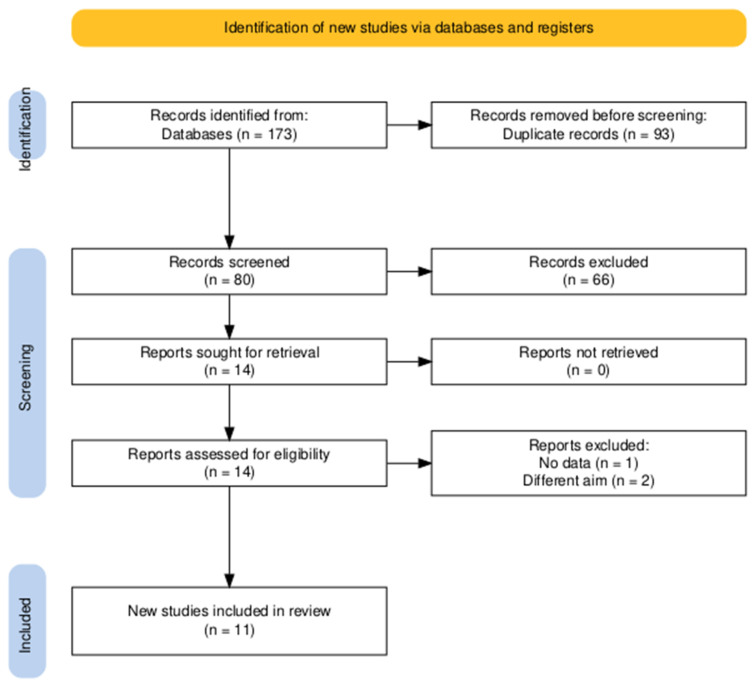
PRISMA flow diagram of searching and screening processes.

The conducted search strategy yielded 173 entries, of which 93 were classified as duplicates. After evaluating the titles and abstracts of the remaining 80 papers, 14 satisfied the criteria for an evaluation of the complete text. In conclusion, 11 publications were deemed suitable for inclusion in the final systematic review [29,30,31,32,33,34,35,36,37,38,39] (Figure 1).

### 3.2. Quality Assessment

According to NOS, three studies were of moderate quality and eight studies were of high quality (Table 1).

### 3.3. Baseline Characteristics

The baseline characteristics of the included studies demonstrate a diverse yet predominantly homogeneous sample of neonates, primarily preterm infants, across multiple high-income countries. All studies employed a cohort design and focused on neonates with or at risk of PDA, with most conducted in Canada (7 out of 11), followed by the USA (3 studies) and Turkey (1 study). Sample sizes varied substantially, ranging from as small as 45 participants (Bischoff 2021) [34] to as large as 307 (Alammary 2022) [37]. The mean gestational ages across studies indicate that the populations were mostly preterm, with gestational ages generally between 22 and 33 weeks. However, two studies (Homedi 2024 and Alammary 2022) [29,37] reported much lower mean ages of 10.3 and 10.1 weeks, respectively, possibly indicating postnatal age. The narrowest age variation was noted in Yadav 2023 (25.0 ± 0.4 weeks) [30], while the widest was seen in Homedi 2024 (10.3 ± 12.5 weeks) [29]. Male representation ranged from 53% to 70% where reported, with most studies showing a slight male predominance. Notably, two studies (EL-Khuffash 2013 and Bischoff 2025) [36,39] did not report sex distribution. The clinical settings also varied slightly, encompassing neonatal intensive care units, perinatal centers, and surgical or interventional contexts related to PDA management, such as ligation or percutaneous closure (Table 2).

The included studies collectively examine the implementation, utility, and clinical impact of TnECHO in the management of neonates—particularly preterm infants—diagnosed with or at risk for PDA and other hemodynamic disturbances.

#### 3.3.1. Utility of TnECHO

A core theme across the studies is the role of TnECHO in enhancing diagnostic precision and informing individualized therapeutic strategies. Several studies, including Jain 2012, Yadav 2023, and Homedi 2024 [29,30,31], demonstrated that the integration of TnECHO into routine care allows for the early identification of infants at high risk of cardiorespiratory instability, particularly in the perioperative context of PDA ligation or closure. Specifically, Yadav 2023 [30] reported a substantial reduction (49%) in PDA ligation rates following the adoption of a standardized TnECHO-based hemodynamic assessment protocol, without any increase in postoperative morbidity—highlighting the modality’s potential to prevent unnecessary invasive interventions.

The predictive utility of TnECHO was further supported by Elsayed 2016 [33], who found that combining TnECHO with biomarkers such as brain-type natriuretic peptide (BNP) and scoring systems like the PDA score at 48–72 h of life could help anticipate the development of significant neonatal morbidities. Similarly, Bischoff 2025 [36] identified a correlation between PDA status, particularly the presence of moderate-to-large left-to-right shunts, and patterns of post-ductal hypotension, further supporting the use of TnECHO for real-time physiological profiling. In addition, Bischoff 2021 [34] contributed a nuanced understanding of systemic perfusion by showing that superior vena cava (SVC) flow remained stable despite increased left ventricular output in the presence of a PDA, raising questions about the reliability of both SVC flow and left ventricular output (LVO) as surrogates for systemic blood flow in this context. These physiological insights have important implications for how clinicians interpret hemodynamic data and make treatment decisions in neonates.

#### 3.3.2. Implementation of TnECHO

From a system and implementation perspective, studies such as EL-Khuffash 2013, Alammary 2022, Papadhima 2018, and Homedi 2024 [29,37,38,39] provided robust evidence that the establishment of a TnECHO consult service within neonatal units significantly influenced clinical decision-making, enhanced diagnostic accuracy, and led to more individualized and timely interventions. These studies reported increased utilization of TnECHO over time and noted that the greatest clinical benefit was observed in cases of high illness severity or complex cardiovascular physiology. Importantly, these services improved interdisciplinary collaboration between neonatologists and pediatric cardiologists, which was highlighted as critical for avoiding misdiagnoses—particularly of structural heart defects—and ensuring high-quality care.

#### 3.3.3. Clinical Impact of TnECHO

Notably, while Papadhima 2018 [38] reported that major cardiac anomalies were consistently identified through TnECHO, some minor congenital defects were missed, reinforcing the need for structured protocols and quality assurance. Mert 2019 [32] provided a broader epidemiological overview, identifying the most common indications for TnECHO as PDA assessment, myocardial function, and systemic blood flow evaluation—corroborating its relevance in daily NICU practice. Finally, Bischoff 2022 [35] examined post-intervention physiology, revealing that percutaneous PDA closure was associated with a drop in left ventricular function and a subsequent risk of oxygenation failure, suggesting that TnECHO can be vital not only in guiding the timing of interventions but also in monitoring for post-procedural complications.

Taken together, the studies offer converging evidence that TnECHO is a valuable bedside tool that improves diagnostic precision, informs therapeutic choices, reduces unnecessary interventions, and enhances overall neonatal care. The modality’s impact is most pronounced when embedded in a multidisciplinary framework supported by structured protocols and ongoing clinician training. Nonetheless, the findings also underscore the importance of recognizing TnECHO’s limitations in anatomical detail and flow estimation, necessitating continued collaboration with pediatric cardiology for comprehensive cardiovascular evaluation (Table 3).

## 4. Discussion

### 4.1. Summary of Findings

The collective findings underscore the pivotal role of TnECHO as a bedside diagnostic and hemodynamic monitoring tool in the management of preterm infants, particularly those with PDA. TnECHO enables real-time, physiology-based assessment of cardiac function, shunt volume, and systemic and pulmonary blood flow, facilitating a deeper understanding of the infant’s cardiovascular status beyond static anatomical evaluations. Its application was associated with improved clinical decision-making, including more precise identification of infants at risk for cardiorespiratory compromise, better characterization of PDA significance, and enhanced selection and timing of therapeutic interventions. Notably, TnECHO-supported management was linked to reduced rates of unnecessary PDA ligations and decreased reliance on empiric treatment approaches, without compromising short-term neonatal outcomes. Furthermore, the adoption of TnECHO services led to measurable changes in clinical practice patterns, characterized by increased echocardiographic utilization, more comprehensive evaluations per patient, and a decline in the need for multiple treatment courses. The tool proved particularly impactful in cases of high illness severity, offering clinicians critical data to support nuanced decisions in complex hemodynamic scenarios. Additionally, its integration into structured consultative services fostered closer collaboration between neonatologists and pediatric cardiologists, enhancing diagnostic accuracy and reducing the likelihood of missed major structural heart defects. While limitations remain—such as operator dependency and potential under-detection of minor anomalies—the overall evidence strongly supports TnECHO as an essential component of individualized, physiology-driven care in the neonatal intensive care unit, with clear benefits for both clinical outcomes and resource optimization. The 2024 American Society of Echocardiography consensus statement emphasizes standardized protocols and competency-based training to ensure quality and safety in TnECHO practice, aligning with our findings on the need for structured implementation and interprofessional collaboration [10].

### 4.2. Clinical Implications

The integration of TnECHO into routine neonatal intensive care offers a significant advancement in the bedside evaluation and management of preterm infants, particularly those affected by PDA and other hemodynamic disturbances. TnECHO provides dynamic, real-time insight into cardiovascular physiology, allowing for a more nuanced understanding of systemic and pulmonary blood flow, shunt significance, and myocardial function. This enables clinicians to tailor interventions based on actual physiological need, rather than relying solely on clinical signs or static anatomical features. One of the most important clinical implications is the potential of TnECHO to reduce unnecessary treatments and procedures. By offering detailed hemodynamic assessments, TnECHO can help identify infants who are unlikely to benefit from pharmacologic or surgical PDA closure, thereby avoiding overtreatment and minimizing the risk of associated complications. This precision-guided approach has been associated with reduced ligation rates and a lower need for multiple treatment courses, without increasing morbidity or instability. TnECHO also supports improved perioperative planning and postoperative surveillance. Its use in the evaluation of infants undergoing PDA closure has revealed patterns of cardiac adaptation and potential post-procedural risks, such as oxygenation failure or compromised ventricular function. Incorporating TnECHO into routine monitoring allows clinicians to detect these complications early and intervene accordingly, potentially improving both short- and long-term outcomes. In terms of healthcare delivery, the establishment of dedicated TnECHO services has been shown to positively influence clinical decision-making and foster interprofessional collaboration, particularly between neonatologists and pediatric cardiologists. This collaborative model enhances diagnostic accuracy—especially in complex or ambiguous cases—and reduces the risk of missing major structural cardiac anomalies. Despite its benefits, the widespread implementation of TnECHO requires thoughtful infrastructure planning, standardized protocols, and sustained clinician training. Its operator-dependent nature and potential for missing subtle anatomical findings underline the continued need for partnership with pediatric cardiology. Future work should focus on refining diagnostic thresholds, validating scoring systems, and evaluating long-term outcomes to ensure the sustainable integration of TnECHO into neonatal care.

The longitudinal TnECHO assessment enhances diagnosis accuracy for diseases that may be clinically obscure or difficult to differentiate, especially in preterm newborns with other comorbidities. This facilitates the modification of PDA care regimens and the individualization of treatment decisions, hence preventing needless exposure to therapeutic medicines that may have adverse side effects [38,40]. A previous study showed that a comprehensive assessment of PDA and physiology-based therapy enhanced outcomes in periviable neonates, indicating its safety even among the most vulnerable neonatal population [41]. Nevertheless, potential adverse effects include strain on infrastructure, heightened maintenance costs for such services, higher management of vulnerable newborns, and an elevated risk of compromising physiological stability.

An ongoing discussion persists regarding which criteria, whether singularly or in combination, most effectively delineate treatment needs. Efforts are underway to develop a grading system that may assist doctors in identifying the demographic that derives the greatest benefit from treatment. The TnECHO era signifies a period during which TnECHO neonatologists employed extensive echocardiographic metrics to detect high-risk infants exhibiting particular hemodynamic traits necessitating intervention. This integrated strategy resulted in an increased number of evaluations conducted per patient and a greater volume of hemodynamic information supplied during the TnECHO period. We hypothesize that the thorough evaluation in the TnECHO era, characterized by more consistent post-treatment echocardiographic examinations, will facilitate improved patient selection for repeated treatment cycles and mitigate unnecessary interventions [29].

#### Prior Literature

The trend of reduced ligation aligns with the existing research, suggesting a lack of advantage associated with significant surgical interventions [42,43]. Yadav et al. [30] discovered that a comprehensive hemodynamic strategy utilizing clinical and echocardiographic metrics facilitates the selection of newborns who may benefit from PDA ligation. Resende et al. [44] showed a decrease in the PDA ligation rate from 10.7% to 5.3% following the implementation of the full TnECHO program in Ontario’s Central East Region. A previous study by Isayama et al. [42] comparing proactive and selective approaches to PDA treatment revealed a lower PDA ligation rate in Japan compared to Canada (1% versus 13%). This discrepancy may be attributed to variations in echocardiography utilization for PDA evaluation, differing ligation criteria, and heterogeneous admission populations and outcome measurements.

### 4.3. Limitations and Recommendations

This study has several limitations that should be acknowledged. Firstly, the review only included cohort studies, which inherently carry a higher risk of bias compared with RCTs. This may limit the strength of the evidence and the ability to infer causality. Secondly, the generalizability of the findings is limited, as most of the included studies were conducted in high-income countries, predominantly Canada and the US. This geographical limitation may not reflect the feasibility or effectiveness of TnECHO in low- and middle-income settings where resource constraints and differences in neonatal care practices exist. Additionally, there was notable heterogeneity in how outcomes were measured across studies, and key data such as long-term outcomes or standardized protocols for TnECHO use were not consistently reported. Furthermore, the observational nature of the included studies increases the possibility of confounding factors influencing the results. Finally, the relatively small sample sizes in some studies may limit the statistical power to detect significant effects or rare complications.

Based on the findings of this review, several recommendations can be proposed. There is a clear need for high-quality, multicenter RCTs to better evaluate the efficacy and safety of TnECHO in the management of PDA in neonates. Future research should aim to include more diverse populations, particularly from low- and middle-income countries, to enhance generalizability and assess the feasibility of implementing TnECHO in varied clinical settings. Standardized protocols for TnECHO training, implementation, and outcome measurement should be developed to ensure consistency across studies and improve comparability. Furthermore, long-term follow-up studies are recommended to assess the impact of TnECHO-guided interventions on neurodevelopmental outcomes and overall neonatal morbidity and mortality. Investment in capacity-building and training programs in neonatal echocardiography, particularly in resource-limited settings, is also essential to ensure equitable access to this promising tool.

## 5. Conclusions

This systematic review highlights the potential of TnECHO as a promising tool in the assessment and management of PDA in neonates, particularly among preterm infants. The findings suggest that TnECHO may offer clinical benefits, such as improved diagnostic accuracy and better-informed therapeutic decisions, although current evidence is limited to observational studies. The predominance of studies from high-income countries underscores the need for broader research efforts to assess the global applicability of this approach. While the results are encouraging, more rigorous research, including RCTs and long-term outcome studies, is needed to establish standardized practices and fully understand the role of TnECHO in neonatal care.

## Figures and Tables

**Table 1:** Quality assessment of retrospective studies using New Castle Ottawa Scale.

Study Name	The Level of Representation of the Affected Cohort (★)	Identification of the Unexposed Cohort (★)	Determination of Exposure (★)	Evidence That the Outcome of Interest Was Absent at the Commencement of the Research (★)	Comparison of Cohorts Based on Design or Assessment (Max★★)	Was the Follow-up Duration Sufficient for Consequences to Manifest? (★)	Evaluation of Results (★)	Assessment of Cohort Follow-up Sufficiency (★)	Quality Level
Jain 2012	★	★	★	★	★★	★	★	★	High
Mert 2019	★	-	★	★	-	★	★	★	Moderate
Elsayed 2016	★	★	★	★	★★	★	★	★	High
Bischoff 2021	★	★	★	★	★★	★	-	★	High
EL-Khuffash 2013	★	-	★	★	-	★	★	★	Moderate
Homedi 2024	★	★	★	★	★★	★	★	★	High
Papadhima 2018	★	★	★	★	★★	★	★	★	High
Alammary 2022	★	-	★	★	-	★	★	★	Moderate
Bischoff 2025	★	★	★	★	★★	-	★	★	High
Yadav 2023	★	★	★	★	★★	★	★	★	High
Bischoff 2022	★	★	★	★	★★	★	★	★	High

**Table 2:** Baseline characteristics of the included studies.

Study ID	Country	Design	Population	Sample Size	Age, Mean (SD), Weeks	Male, *n* (%)
Jain 2012	Canada	Cohort	Preterminfants undergoing PDA ligation	52	25.5 (2)	28 (53.8)
Mert 2019	Turkey	Cohort	Neonates in the intensive care unit	186	33.1 (4.1)	106 (57)
Elsayed 2016	Canada	Cohort	Preterm infants with PDA	71	28 (1.6)	37 (53)
Bischoff 2021	USA	Cohort	Preterm infants with PDA shunt	45	25.45 (2.11)	25 (55.6)
EL-Khuffash 2013	Canada	Cohort	Neonates in the intensive care unit	199	27.9 (3.4)	NR
Homedi 2024	Canada	Cohort	Neonates in the intensive care unit	275	10.3 (12.5)	153 (55.6)
Papadhima 2018	Canada	Cohort	Neonates in the perinatal center	268	26.4 (3.4)	156 (59)
Alammary 2022	Canada	Cohort	Neonates in the intensive care unit	307	10.1 (1.7)	195 (63)
Bischoff 2025	USA	Cohort	Extremely preterm infants during the first two postnatal weeks	141	22–28	NR
Yadav 2023	Canada	Cohort	Preterm infants who underwent PDA ligation	69	25 (0.4)	43 (62.3)
Bischoff 2022	USA	Cohort	Preterm infants ≤ 2 kg undergoing percutaneous PDA closure	50	24.9 (1.8)	35 (70)

PDA: patent ductus arteriosus.

**Table 3:** Aim and summary of findings of the included studies.

Study ID	Aim	Summary of Findings
Jain 2012	To investigate the value of TnECHO in predicting cardiorespiratory instability after PDA ligation, and to evaluate the impact of TnECHO-directed care.	TnECHO facilitates early detection of infants at greatest risk for subsequent cardiorespiratory deterioration.
Mert 2019	To determine the frequency of use of TnECHO, and the patient characteristics and indications.	TnECHO is often used, and it can be a useful tool in guiding treatment. Assessment of PDA, myocardial performance and systemic blood flow are the most common indications for use.
Elsayed 2016	To compare PDA diameter, PDA score and BNP measurements at 48–72 h of life, for prediction of neonatal morbidities and the pateints were studied using TnECHO.	Comprehensive PDA evaluation at 48–72 h of age using PDA score, TnECHO and BNP measurements may predict the subsequent occurrence of adverse outcomes and may be useful to define the PDA treatment threshold.
Bischoff 2021	To investigate the relationship between LVO and RVO, and SVC flow, measured concurrently, in preterm infants according to directionality of PDA shunt using comprehensive TnECHO.	SVC flow was maintained irrespective of increased pre-ductal cardiac output in patients with left-to-right shunt. This may reflect autoregulation of cerebral perfusion and/or concerns with the methodology of SVC flow estimation. While it is physiologically clear that LVO is not a reliable measure of post-ductal systemic blood flow, the contrary should also not be interpreted; specifically, that SVC flow is a superior measure of systemic blood flow in the presence of a PDA.
EL-Khuffash 2013	To characterize the effect of TnECHO program on decision-making in a tertiary level unit.	The evolution of the TnECHO consultative service has led to clinical practices changes, which appear to be beneficial. The practice of TnECHO needs to be properly regulated within a secure infrastructure where there is close collaboration between trained neonatologists and pediatric echocardiography laboratories or pediatric cardiologists with expertise in echocardiography.
Homedi 2024	To compare PDA-related hemodynamic information and PDA treatment decisions before and after introduction of TnECHO service.	With the implementation of the TnECHO service, increased echocardiographic evaluations per patient were completed with availability of more comprehensive hemodynamic information about PDA. PDA treatment rates were similar in the two epochs but need for multiple courses were less in TnECHO era.
Papadhima 2018	To describe the utilization and study the factors associated with the impact on clinical management of a new TnECHO consultation service in a perinatal center.	TnECHO consult service demonstrated an increasing utilization and a significant impact on clinical management over time especially for non-PDA indications and in situations of high-illness severity. Although, all major cardiac defects were identified, some minor congenital defects were missed by TnECHO.
Alammary 2022	To evaluate the impact of TnECHO service on patient management in the neonatal intensive care units in Winnipeg, Canada.	TnECHO service in the neonatal intensive care unit in Winnipeg guided the clinical management in a significant proportion of patients who received the service thereby enhancing clinical care. The collaboration between the TnECHO team and pediatric cardiology provided high level efficient service avoiding missed diagnosis of major structural heart defects.
Bischoff 2025	To evaluate pre- and post-ductal blood pressure in preterm infants according to PDA status obtained from TnECHO.	Blood pressure patterns measured by TnECHO varied according to PDA status. Post-ductal hypotension was more common with a moderate-high volume shunt. PDA status in this population may be the strongest influencer of blood pressure variability.
Yadav 2023	To evaluate the preoperative assessment impacts of the hemodynamic significance of PDA using TnECHO on PDA ligation rates and neonatal outcomes.	Incorporating TnECHO into a standardized hemodynamic assessment program, a 49% reduction was demonstrated in PDA ligation rate without any increase in postoperative cardiopulmonary instability or short-term neonatal morbidities in a cohort of very low birth weight infants.
Bischoff 2022	To evaluate TnECHO characteristics and the clinical course of preterm infants ≤ 2 kg undergoing percutaneous PDA closure.	Percutaneous PDA closure leads to a reduction in echocardiography markers of left ventricular systolic/diastolic function. Post closure cardiorespiratory instability is characterized primarily by oxygenation failure and may relate to impaired diastolic performance.

TnECHO: targeted neonatal echocardiography; PDA: patent ductus arteriosus; BNP: brain-type natriuretic peptide; LVO: left ventricular output; RVO: right ventricular output; SVC: superior vena cava.

## Data Availability

The datasets analyzed during the current study are available from the corresponding author upon reasonable request.
